# Differences in pragmatic communication skills of adults with intellectual disabilities and dual diagnoses

**DOI:** 10.3389/fpsyt.2023.1072736

**Published:** 2023-02-02

**Authors:** Mirjana Djordjevic, Nenad Glumbić, Branislav Brojčin, Slobodan Banković, Vesna Žunić Pavlović

**Affiliations:** Faculty of Special Education and Rehabilitation, University of Belgrade, Belgrade, Serbia

**Keywords:** clinical population, comorbidity, pragmatics, intellectual disabililties, dual diagnosis

## Abstract

**Introduction:**

Pragmatics includes a set of skills related to language structure and meaning that allow the speaker to use the language appropriately and in accordance with different communication situations. The aim of this research was to determine the differences in pragmatic communication skills of adults with intellectual disabilities, dual diagnoses, and typical development, and to determine the effects of gender, age, the level of intellectual functioning and speech comprehension on their achievements on two assessment instruments.

**Methods:**

The sample included 180 adults (60 typically developing participants, 60 with intellectual disabilities, and 60 participants with dual diagnoses). We used two instruments to assess pragmatic communication skills – Communication Checklist – Adult, CC-A, and the Assessment Battery for Communication, ABaCo. In order to test the differences between the three groups of participants, we used canonical discriminant analysis.

**Results:**

Discriminant analysis revealed two significant canonical functions. Function one (speech comprehension and the level of intellectual disability, social engagement, and paralinguistic scale) differentiates between typically developing participants and participants with dual diagnoses the most. The second canonical function (language structure, linguistic scale, paralinguistic scale, extralinguistic scale, and context scale) differentiates between participants with intellectual disabilities and participants with dual diagnoses the most. According to the results, age did not affect pragmatic achievements.

**Discussion:**

Pragmatic skills are very complex, and different instruments measure different dimensions of these abilities. The results of this research lead to the conclusion that we can differentiate between the pragmatic abilities of typically developing people, people with intellectual disabilities, and those with dual diagnoses with the help of the ABaCo battery and the CC-A questionnaire.

## 1. Introduction

Pragmatic communication involves using different expressive means to convey and interpret meaning in a specific context (1). The range of such expressive means is vast. Apart from purely linguistic ones (semantics, syntax, speech acts, metapragmatics, discourse organization, etc.), they also include different forms of non-verbal communication, i.e., paralinguistic (e.g., production and comprehension of irony and deception) and extralinguistic (e.g., body language and facial expression) communication skills (2). Pragmatic skills are necessary for producing and understanding meaning in a specific social context.

The contexts and situations in which language is used are so diverse that it is difficult to set boundaries. Contextual knowledge includes not only the present contextual situation but also what happened in the past, interlocutors’ relationships, assumed knowledge, etc. (1, 3). Thus, pragmatic communication is very complex, and most research studies deal with one or a small number of pragmatic phenomena. Thus, pragmatic communication can be evaluated in different ways. Apart from the division regarding whether the instrument is standardized, methods of collecting data in pragmatics may vary depending on whether discourse analyses, rating scales, analogue tasks, or test batteries are used. Bearing in mind that different instruments measure different domains of pragmatics and that each assessment approach has its drawbacks and advantages, it is advisable to combine various instruments when collecting data on pragmatic competencies (4).

Speakers are most often spontaneously guided by implicit rules of pragmatic communication, which we only become aware of when they are broken (5). Pragmatic outbursts are, of course, very common in everyday communication. However, in individuals with a pragmatic disorder, the deficits in pragmatic competence are of such scope and intensity that they affect social relations and quality of life (6). Pragmatic language disorder can be viewed as a separate clinical entity or as one of the symptoms of other clinical conditions (7). In the clinical population, pragmatic disorders have most frequently been examined in adults with traumatic brain injury, damage to the left or right hemisphere, and schizophrenia e.g., (8–12) and in children with neurodevelopmental disorders such as attention deficit hyperactivity disorder and autism e.g., (13–15).

In people with intellectual disabilities (ID), pragmatic disorders have mainly been examined in children and adolescents. Research has primarily focused on determining specific profiles of pragmatic functioning or individual pragmatic phenomena in participants with different syndromes (16). A similar research trend is present in examining pragmatic communication in adults with Fragile X syndrome and Williams syndrome (17), Down syndrome (DS) and ID of unknown etiology (18).

A comprehensive assessment of the pragmatic abilities of adults with schizophrenia indicates that their deficits go far beyond the linguistic aspects of pragmatic communication and into the area of extralinguistic, paralinguistic, contextual, and conversational aspects of communication (19). Linscott (20) believes that pragmatic deficits in people with schizophrenia are a secondary consequence of general cognitive decline, measured by determining a discrepancy between the present and premorbid intelligence quotient. Participants with the so-called dual diagnoses (DD), who have a comorbid psychiatric condition in addition to ID, represent a special category. One-third of adults with ID are considered to have comorbid psychiatric disorders e.g., (21, 22). However, research studies on the pragmatic abilities of people with DD are scarce and, as a rule, indicate more significant pragmatic deficits in people with DD compared to participants with ID. Participants with ID are better at irony comprehension tasks than participants with DD (23). It has also been shown that the presence of DD and the level of ID independently affect participants’ paralinguistic abilities (24). People with DD have more significant procedural discourse impairments than participants with ID, manifested in their inability to consider their communication partner’s needs while a game or task is being described (25).

In addition to general cognitive ability, structural language abilities are crucial in the developing of pragmatic communication. From a developmental perspective, acquiring pragmatic skills is closely related to mastering other aspects of language structure (1). Thus, Martin (16) points out the necessity to equalize children according to mental age and language abilities when determining syndrome specificities in the field of pragmatics to ensure that basic language competencies are similar in different subsamples. Panzeri et al. (26) indicate a relation between general language competence and some paralinguistic skills in participants with DS. Research conducted using a sample of 10 adults with ID, and DD shows a positive correlation between speech comprehension ability and pragmatic competence (27).

Participants’ age is one of the possible factors that can influence pragmatic communication. By examining the ability to understand sarcasm and teasing in videos shown to a sample of typically developing (TD) participants, 18–76 years of age, it was found that older participants had considerable difficulties distinguishing between literal and non-literal meaning (28). It has been observed that age negatively affects the general population’s ability to keep to a topic, discourse coherence, and the ability to understand metaphors, idioms, proverbs, and jokes (29). A recent study shows that healthy participants over 65 years old have significantly poorer paralinguistic, extralinguistic, and contextually appropriate speech comprehension and production skills than participants 20–40 years of age (30). To our knowledge, the differences between a wide range of pragmatic abilities in adults with ID and DD of different ages have not been examined.

## 2. Aim

The aim of this article was to determine the differences in achievements on two instruments for assessing pragmatic communication skills in adults with ID, DD and TD, and to determine the effects of gender, age, the level of intellectual functioning and speech comprehension on their achievements on two assessment instruments.

## 3. Materials and methods

### 3.1. The sample

The sample included 180 people, equal by gender (χ^2^ (1) = 0.022, *p* > 0.05), age range 20–56 years old (M = 28.45, SD = 8.59). The sample was divided into three groups – a group of participants with ID (*n* = 60), a group with DD (*n* = 60), and a control group (*n* = 60). One-factor analysis of variance found statistically significant differences between the subsamples with regard to age (F(2,177) = 41.890, *p* < 0.01). The subsequent Scheffe test determined that participants with DD and ID were equal with regard to age (*p* > 0.05), and that the differences existed between the control group (M = 21.70, SD = 1.453) and the participants with ID (M = 32.95, SD = 8.833, *p* < 0.01) and DD (M = 30.70, SD = 8.494, *p* < 0.01). Due to the detected differences, age was considered a control variable in further analyses. In both assessed groups (ID and DD), half of the participants lived in family homes and the other half in an institution. No statistically significant difference was determined between these two subsamples with regard to the place of living (group of participants with ID – *U* = 450.00; *Z* = 0.000; *p* > 0.05; group of participants with DD – *U* = 449.50; *Z* = 0.000; *p* > 0.05).

An additional grouping factor was created by identifying sub-groups with mild and moderate ID in both the ID and DD groups in order to test the effect of ID levels.

There were 25 participants with mild and 35 with moderate ID in both the group with ID and the group with DD.

All participants with ID were diagnosed in childhood. They were re-diagnosed and assessed by a psychiatrist after being admitted to a social welfare institution. There were 10 participants with Down syndrome in the ID group. All participants with Down syndrome were in the ID group with no comorbid psychiatric diagnosis, functioning at the level of moderate ID, and did not use any medications. The etiology was unknown in all other participants with ID. There were no participants with autism spectrum disorders in both groups with ID and DD. The participants with DD (according to their personal records) mainly exhibited the symptoms of schizophrenia spectrum disorders (e.g., delusions, hallucinations, abnormal motor behavior, negative symptoms, and disorganized speech and thoughts).

Data on the level of intellectual functioning and DD were taken from their personal records, with previously obtained informed consent from the participants and their parents or caregivers. Raven’s progressive matrices were used in this research to determine the level of intellectual functioning as a control variable.

In forming all groups of participants, exclusion criteria were the following: hearing and visual impairment, bilingualism, traumatic brain injury, and neurosurgical interventions.

Table 1 is a descriptive presentation of participants’ achievements on the Peabody test for assessing speech comprehension, expressed through the mean, standard deviation, and standard error.

**TABLE 1:** The structure of subsamples with regard to speech comprehension.

	Subsample	*n*	M	SD	SE
Speech comprehension ability	ID	60	95.22	44.39	5.731
	DD	60	78.12	43.38	5.600
	Control group	60	219.92	3.58	0.462

*n*, number of participants; M, mean; SD, standard deviation; SE, standard error.

One-factor analysis of variance found statistically significant differences between the examined groups with regard to speech comprehension (F(2,177) = 279.014, *p* < 0.01). The subsequent Scheffe test determined that there were differences between participants from all groups. Thus, the participants from the control group had significantly better results than the participants with ID (*p* < 0.01) and the participants with DD (*p* < 0.01). Also, speech comprehension ability was more developed in participants with ID than in those with DD (*p* < 0.05). Due to the obtained differences, speech comprehension was considered a control variable.

All participants with DD were on medication therapy, while those with ID and the participants from the control group did not use psychotropic medications.

### 3.2. Assessment instruments

Communication Checklist – Adult, CC-A (31) is a questionnaire (rating scale) for assessing the pragmatic communication skills of adults who are at least 17 years of age and have developed speech (people whose speech is on the sentence level rather than on the level of individual words). The checklist includes 70 items that the informants grade 0–3 depending on the manifestation degree of the examined behavior. Behaviors that do not occur or occur less than once a week are graded 0, behaviors that occur once a week are graded 1, behaviors manifested once or twice a day are graded 2, while those occurring several times a day or always are graded 3. The first 50 items assess the degree of participants’ communication difficulties, while the remaining 20 items refer to speech-language abilities. After administering CC-A, raw, scaled, and composite scores can be calculated (raw scores were used for the purpose of this research). Raw scores are obtained by re-coding previous scores referring to potentials so that the entire scale is negative (a higher score indicates poorer pragmatic communication skills). This checklist assesses three areas: language structure (phonological organization, grammar, and vocabulary), pragmatic abilities (coherence, inadequate initiation of communication, stereotyped speech, and interests), and social engagement (communication context, non-verbal communication, and social interactions). The Language Structure subscale is used to assess the linguistic aspects of language reflected in speech, syntax, and semantics. The Pragmatic Skills subscale involves assessing expressive pragmatic behaviors, while the Social Engagement subscale can be used to evaluate non-verbal aspects of communication and participants’ interests. Whitehouse et al. (32) state that Cronbach’s alpha is high at α = 0.90. Table 2 shows Cronbach’s alpha for this scale for all three groups of participants. Data was collected from therapists (special educators) who knew the participants for at least six months and had direct contact with them on a daily basis.

**TABLE 2:** Cronbach’s alpha of ABaCo and CC-A.

		Cronbach’s alpha			
Scale	Subscale	Whole sample	control	ID	DD
CC-A	Language Structure	0.963	0.838	0.923	0.920
	Pragmatic Skills	0.914	0.831	0.897	0.856
	Social Engagement	0.959	0.907	0.925	0.884
ABaCo	Linguistic	0.703	0.454	0.454	0.701
	Extralinguistic	0.715		0.630	0.681
	Paralinguistic	0.720	0.360	0.544	0.578
	Context Scale	0.570	0.519	0.336	0.569
	Conversational	0.771		0.762	0.786

The Assessment Battery for Communication, ABaCo (33), is a comprehensive clinical instrument for evaluating pragmatic communication skills. The instrument includes five scales: Linguistic, Extralinguistic, Paralinguistic, Context, and Conversational. Within each scale, except the Conversational one, tasks are grouped into two subcategories – for assessing comprehension and production abilities. There are 172 items in total, with 100 items given as short videos, while 72 are direct items within which examiners ask a question and participants are their interlocutors. In tasks involving videos, examiners show a video scene and then ask a question related to the communicative interaction in the video. The video scenes last 20–25 seconds, and the number of words in the video materials ranges from five to nine. Each correct answer is graded 1 and incorrect 0. Depending on the scale and the task type, the maximum number of points for one task will differ. The raw score for the whole battery and the scores for each subscale are obtained by adding up all points. The scoring is done according to the recommendations of the battery authors (33) and the authors of a research study in which the Scale was used (34). According to the scale authors, the entire battery has high internal consistency (33). Table 2 shows Cronbach’s alpha for this scale for all three groups of participants.

The data presented in Table 2 indicates that the reliability of both applied pragmatic abilities assessment scales is similar in all three groups, except the ABaCo Linguistic scale (it is lower in the control group and the ID group) and the Paralinguistic scale (it is low in all three groups). Also, the Extralinguistic and Conversational scales have no reliability in the control group since all participants had the maximum score on most questions, so there is practically no variability.

Raven’s progressive matrices (35) were used to determine the level of intellectual functioning as a control variable. This instrument consists of non-verbal tasks for measuring general intelligence. The tasks within this test are organized as “patterns” so that one segment is always missing. Participants are expected to recognize the rule of the pattern and select the one that is missing from several offered. The used version of matrices includes 60 tasks organized in five sets. The tasks are arranged by difficulty, and the sets are organized according to topics: completing continuous patterns, discovering analogies between pairs of figures, changing patterns progressively, rearranging figures, and breaking figures into parts. The reliability coefficient determined by the odd-even method is high at.96, while the test-retest reliability is somewhat lower (0.88).

The Peabody Picture Vocabulary Test, PPVT–4 (36), was used to assess speech comprehension as a control variable. The items are grouped into 19 categories with 12 words each. The total number of words is 228. Out of four given pictures, participants are expected to show the one that corresponds to the spoken word. The Peabody test has high internal consistency ranging from 0.92 to 0.98. Electronic version of this test was used in this research. The participants were shown pictures on a computer screen. Correct and incorrect answers were entered in a form. The testing was stopped when a participant had eight incorrect answers in one set. The raw score was obtained by subtracting the number of incorrect answers from the total number of items.

### 3.3. Ethics statement

The studies involving human/animal participants were reviewed and approved by Ethics Committee of the Faculty of Special Education and Rehabilitation from University of Belgrade.

## 4. Results

### 4.1. Control variables – IQ measures and speech comprehension

Having in mind that IQ measures (Raven measures) can correlate with speech comprehension (PIBODI), we tested this and obtained a high positive correlation between the two (r = 0.88, *p* < 0.01). Since we planned to control both of these measures, a high correlation between them might lead to multicollinearity. In order to avoid it, we standardized measures on Raven and PIBODI, averaged them, and used this averaged measure as a covariate in further analyses (we will call it *ability*).

### 4.2. Dimensions of communication ability instruments

On the other hand, dimensions of communication ability instruments, CC-A and ABaCo, do correlate, but correlations are mainly below 0.7, with only three of them showing values up to 0.76 (Table 3).

**TABLE 3:** Correlation coefficients between AbaCo and CC-A scales (*N* = 180).

Scales ABaCo – CC-A		Language structure	Pragmatic skills	Social engagement
Linguistic	*r*	-0.580**	-0.475**	-0.596**
	*p*	0.000	0.000	0.000
Extralinguistic	*r*	-0.694**	-0.491**	-0.718**
	*p*	0.000	0.000	0.000
Paralinguistic	*r*	-0.718**	-0.526**	-0.759**
	*p*	0.000	0.000	0.000
Conversational	*r*	-0.501**	-0.304**	-0.569**
	*p*	0.000	0.000	0.000
Context Scale	*r*	-0.670**	-0.451**	-0.626**
	*p*	0.000	0.000	0.000

*r* = correlation index; *p* = significance. **Correlation is significant at the 0.01.

In addition, we applied exploratory factor analysis with a principal component method and varimax rotation of axis, which resulted in a two-factor solution explaining 73% of the variance in total (42.9 and 30.1%). As shown in the rotated structure matrix (Table 4), the first factor is mainly saturated by AbaCo scales, while the second factor mainly correlates with CC-A scales. Only two AbaCo scales, linguistic understanding and production, show higher saturation on CC-A factor, which is expected since these two scales more directly refer to language.

**TABLE 4:** Structure matrix for AbaCo and CC-A scales.

Instrument Scales		Factor 1 (AbaCo)	Factor 2 (CC-A)
CC-A	Language structure	-0.556	-0.672
	Pragmatic skills		-0.735
	Social engagement	-0.579	-0.658
ABaCo	Linguistic comprehension		0.814
	Linguistic production		0.829
	Paralinguistic comprehension	0.783	0.435
	Paralinguistic production	0.738	0.402
	Conversational	0.764	
	Extralinguistic comprehension	0.820	0.378
	Extralinguistic production	0.807	0.373
	Context Scale comprehension	0.820	
	Context Scale production	0.746	0.303

Having all this in mind, correlations below 0.7 or 0.76, with only three of them showing values up to 0.76, and two separate factors extracted from AbaCo and CC-A scales, we decided to use both of these measures as separate measures of communication ability.

### 4.3. Differences between the examined groups on CC-A and ABaCo battery scales

Table 5 shows the mean values of achievements on ABaCo battery and CC-A scales.

**TABLE 5:** Achievements of all three groups of participants on the ABaCo battery and CC-A scales.

Instrument scales		Subsample	*n*	M		SD	SE
ABaCo	Linguistic	ID	60	61.52		14.87	1.92
		DD	60	62.02		15.47	1.99
		Control group	60	85.20		3.50	0.45
	Extralinguistic	ID	60	46.13		18.10	2.33
		DD	60	33.95		21.20	2.73
		Control group	60	77.33		5.09	0.65
	Paralinguistic	ID	60	18.98		5.52	0.71
		DD	60	15.22		6.02	0.77
		Control group	60	30.62		1.88	0.24
	Conversational	ID	60	21.07		1.85	0.24
		DD	60	20.23		2.18	0.28
		Control group	60	23.93		0.25	0.03
	Context scale	ID	60	17.23		9.63	1.24
		DD	60	14.35		8.72	1.12
		Control group	60	27.38		5.29	0.68
CC-A	Language structure	ID	60	24.03	14.46		0.87
		DD	60	24.72	15.51		2.00
		Control group	60	2.15	3.70		0.48
	Pragmatic skills	ID	60	17.42	10.95		1.41
		DD	60	20.97	11.90		1.54
		Control group	60	4.38	5.36		0.69
	Social engagement	ID	60	32.43	14.31		1.85
		DD	60	38.90	16.46		2.13
		Control group	60	5.75	7.50		0.97

*n*, number of participants; M, mean; SD, standard deviation; SE, standard error.

We used canonical discriminant analysis to test differences between the three groups of participants (control, ID, and DD) and to control ability measures (Raven and PIBODI), gender, and age. This analysis defines a function as a linear combination of predictors which best discriminates three groups. Apart from the control variables (ability, gender, and age), we added measures of communication, AbaCo, and CC-A scales as predictors.

Discriminant analysis revealed two significant canonical functions (Table 6). Based on these two functions, 87.2% of original grouped cases were correctly classified, which indicates a high success rate.

**TABLE 6:** Significance of canonical functions.

Test of Function(s)	Wilks’ Lambda	Chi-square	*df*	*P*	*Rho*
1 through 2	0.044	537.345	22	0.000	0.962
2	0.588	91.199	10	0.000	0.642

*df*, the degrees of freedom; *p*, significance; *rho*, rank correlation coefficient.

Based on the structure (correlations of each variable with a function) and canonical coefficients (weights), we can detect the structure of both canonical functions or detect which variables are most effective in differentiating three groups (control, ID, and DD). The structure should be above 0.3, and it should be in the same direction as a canonical coefficient (in order to avoid suppression effects).

We can see that function 1 (Table 7) is constituted by ability (Raven and PIBODI), social engagement (CC-A), and paralinguistic scale (AbaCo). This function differentiates between the control and the DD group the most, and the difference between these two groups is almost eight standard deviations, which is considered a very large effect. The difference between the ID and DD groups is smaller than one standard deviation, so the ID group score is closer to DD than to the control group. Having in mind that higher scores in CC-A indicate lower communication abilities, we can see that the control group has higher ability (Raven and PIBODI), communication, and pragmatic skills (social engagement and paralinguistic) than both ID and DD groups, while DD group has lowest scores on these three scales.

**TABLE 7:** Structure of canonical functions.

	Function 1		Function 2	
	Coefficient	Structure	Coefficient	Structure
Gender	0.164	0.008	-0.040	0.128*
Age	-0.118	-0.188*	0.151	0.147
Ability (Raven and PIBODI)	1.093	0.875*	-0.443	0.164
Language structure	0.104	-0.304	-0.820	-0.634*
Pragmatic skills	-0.039	-0.215	0.148	-0.232*
Social engagement	-0.041	-0.326*	0.261	-0.293
Linguistic	0.182	0.287	0.365	0.402*
Extralinguistic	-0.259	0.346	0.325	0.477*
Paralinguistic	0.312	0.413*	0.080	0.409*
Context scale	-0.365	0.235	0.503	0.569*
Conversational	0.094	0.261*	-0.327	-0.077

*Correlation is significant at the 0.05.

The second canonical function (Table 7) is constituted by language structure (CC-A), linguistic scale (AbaCo), paralinguistic scale (AbaCo), extralinguistic scale (AbaCo), and context scale (AbaCo).

This function differentiates between the ID and the DD group the most, and the difference between the two groups is around 2 standard deviations, which is considered a large effect (Table 8). The control group is located between ID and DD groups on this function scores. We can see that the ID group has higher communication (language structure) and pragmatic skills (linguistic, paralinguistic, extralinguistic, and context scale) than the DD group, while the control group is located between the previous two.

**TABLE 8:** Canonical functions centroids on three groups.

Group	Function	
	1	2
Control	4.907	-0.124
ID	-2.003	1.072
DD	-2.904	-0.948

## 5. Discussion

The aim of this paper was to determine the differences in pragmatic skills of adults with ID, DD, and TD on the ABaCo battery and CC-A questionnaire, and to determine the effects of gender, age, the level of intellectual functioning and speech comprehension on their achievements on these two instruments.

### 5.1. Differences between participants with disabilities and TD participants on CC-A and ABaCo

Canonical discriminant analysis singled out two functions that differentiate between the examined groups. Speech comprehension and the level of intellectual functioning, integrated into the “ability” variable, together with the achievements on the Social Engagement scale (CC-A) and the Paralinguistic scale (ABaCo), comprised the first discriminant function differentiating TD participants from those with disabilities (especially the participants with DD). The “ability” variable had the biggest contribution to this discriminant function, which was expected since ID is defined by the below-average level of intellectual functioning (37), while receptive speech is also sometimes taken as a measure of intellectual functioning e.g., (38, 39). Furthermore, our results indicate that the level of paralinguistic abilities distinguishes between the mentioned groups, which is in line with the results of other studies in which participants with schizophrenia (19, 38, 40), and participants with ID (41–43), had worse results than TD participants in this domain of pragmatics. Searching for predictors of worse achievements in identifying and producing emotions in adults with ID, Calić et al. (44) found that receptive language skills were a significant predictor of paralinguistic comprehension of emotions. Other authors also point out the significance of receptive vocabulary, especially the one related to naming emotions in emotion recognition tasks in children with Down syndrome (45). Some studies suggest that there is a link between recognizing emotions and the level of intellectual functioning, i.e., that the difficulties in recognizing emotions increase with the level of ID (46). With regard to that, the significance of difficulties in processing information related to a lower level of intellectual functioning is emphasized, such as memory and attention deficits, imagination, and dealing with static and ambiguous stimuli (47). However, Scotland et al. (42) believe that there is still no clear evidence that a cognitive-intellectual disability can fully explain the deficits in recognizing emotions, and that methodological variations between studies limit the possibility of a reliable interpretation of the causes of these deficits in adults with ID.

Achievements on the Social Engagement scale (CC-A) contribute to the first discriminant function to a lesser degree than the previously mentioned variables. The scale includes a number of items that also refer to paralinguistic communication (highly correlating with the Paralinguistic scale; see Table 3), although it is not used for a more comprehensive assessment of this communication aspect, which could at least partly explain its significance and a smaller contribution to the first discriminant function. This scale generally detects unusual pragmatic models and a passive communication style, with no tendency to get involved in social interaction. Both groups of participants with disabilities had lower achievements on this scale than the TD group. This result may be the consequence of the cognitive and linguistic characteristics of participants with disabilities, as well as environmental factors. Smith et al. (48) point out that the level of ID, social participation, and living in an institution are strong predictors of communication difficulties in adults with ID. Half of our participants with ID and DD live in an institution, and it is stated that the interactions of people with ID, even when they live in a community, are mostly limited to their family members or other people with ID (49). It is possible that the lower achievement on the Social Engagement scale in people with disabilities, compared to TD participants, is related to limited social experience, which leads to a smaller initiative for participating in social interactions.

Interestingly, neither linguistic communication aspects nor extralinguistic (ABaCo) and Pragmatic Skills (CC-A) define the differences between groups with disabilities and TD participants in either of the two scales. It is possible that paralinguistic skills are more evolution-determined (50), while other domains of socio-communication functioning are under a stronger influence of the environment. Although both extralinguistic and paralinguistic aspects of communication belong to non-verbal aspects of communication, they do not belong to the corpus of abilities defining the difference between TD participants and those with disabilities. A possible explanation could be the fact that, from a developmental aspect, gestures are the precursors of language development e.g., (51–53). After the appearance and further development of verbal production, extralinguistic signs have an additional function to follow the verbal expression and/or to substitute speech (54). Furthermore, there are some specificities regarding the use of extralinguistic elements in the population of people with ID. With age and the increase in linguistic production, extralinguistic production decreases in the TD population, while it remains at the same level in people with ID. It has also been found that, with age, people with ID use gestures more often to initiate social interaction (55).

Although studies on TD people have found that older participants have greater difficulties in some aspects of pragmatic competence (6, 18), this was not confirmed in our research. Participants’ age had no effect on the differences between the participants on any of the applied instruments or their scales. However, although the participants’ age range was 20–56, it is possible that it was not wide enough to detect the changes that occur with age. It is stated that there is little evidence that cognitive decline occurs before 60 years of age (56), that overall language ability remains preserved with age, and that some language aspects are stable up to the age of 70 (57). Contrary to the results of this research, by examining the pragmatic skills of TD participants, Hilviu et al. (30) found that the younger group of participants (20–40 years of age) was more successful on some ABaCo scales than the two older groups. However, in that research, older groups included participants over 65 years of age.

Gender also had no discriminant significance. This is in accordance with other studies that found no effect of gender on specific aspects of pragmatics in TD participants (46, 58), participants with schizophrenia (59), and participants with ID (44).

### 5.2. Differences in achievements of participants with ID and participants with DD on CC-A and ABaCo

The results of the second discriminant function indicate that the participants with DD differed from those with ID on a large number of both linguistic and non-linguistic aspects of pragmatics – Language Structure (CC-A), Linguistic, Paralinguistic, Extralinguistic and Context scale (ABaCo) – with gender, age, and ability having no significant influence in differentiating these two groups. The participants with DD had lower achievements than those with ID in all examined aspects. The results are in line with the findings obtained in people with preserved intelligence who have schizophrenia, according to which the greater severity of the symptoms, but not the level of intellectual functioning, is associated with poorer pragmatic abilities (19). The same research found that the participants with schizophrenia were significantly worse than the control group of TD participants in all examined aspects of the ABaCo battery. Matson et al. (60) also report significantly poorer socio-communication skills in adults with mild and moderate ID who have more pronounced psychopathological symptoms.

With regard to the factor analysis, according to which the Linguistic scale (ABaCo) and Language Structure (CC-A) belong to the same factor (they examine a similar construct), it is not surprising that the differences between ID and DD participants in linguistic aspects of pragmatics were detected on both instruments. Although some researchers have not found a relation between the level of intellectual functioning and a wider range of pragmatic skills e.g., (19), the results of other studies suggest that impairment in intellectual functioning generally is a significant predictor of poorer achievements on language tests in people with schizophrenia. Poor language achievement, especially regarding higher-order semantic deficits, is also associated with formal thought disorder (a relatively common symptom of schizophrenia) (61). Similarly, one meta-analytical study found a relation between formal thought disorder (disorganized speech) and semantic processing, as well as between disorganized speech production and cognitive impairments (executive functions deficit) (62). Furthermore, Bakken et al. (63) indicate that adults with ID and a comorbid psychiatric disorder may exhibit disorganized linguistic production characterized by incoherent and poor speech expression, confusion, discomfort, and frustration caused by their interlocutor’s poor understanding, as well as significantly reduced or absent initiative in conversation.

Researchers (64, 65) also found poorer achievements in extralinguistic communication aspects in people with schizophrenia but with averge intellectual functioning. They explain this by these people’s reduced sensitivity, lack of response to gestures, and delusions in communication contributing to misinterpretation, distorted perception, and biased explanation of gestures. Parola et al. (40) have found that, after linguistic irony and violating the Gricean maxims, extralinguistic deceitful and sincere communicative acts are the most relevant pragmatic phenomena in distinguishing people with schizophrenia from healthy participants. These people have an impaired ability to understand emotional semantic content (66). In addition to difficulties in understanding and producing affective prosody and facial expressions, they also have problems with non-affective paralinguistic signs, such as basic speech acts expressed by paralinguistic means (19). Pawełczyk et al. (67) explain extralinguistic and paralinguistic dysfunctions in people with schizophrenia by processing disorders in the right hemisphere of the brain, which can potentially cause serious problems in social communication.

In addition to Language Structure, i.e., linguistic aspects of pragmatics, the achievements on the Context scale also had a relatively large contribution to the second discriminant function. In our research, the participants with DD had greater difficulties than those with ID in adapting their communication to specific contexts (different social situations or communication partners). Matson et al. (60) also indicate such difficulties in participants with mild and moderate ID and comorbid psychiatric disorders, stating that unpleasant and/or bizarre comments can characterize the socio-communicative functioning of these people, while they show a tendency toward unfounded attributions and blaming others in understanding other people’s verbal statements. Their verbal production can also be characterized by elements of verbal aggression. Furthermore, schizophrenia is characterized by specific difficulties in social cognition. They can be manifested in problems related to perceiving and processing social and emotional signals, explaining and finding the causes of positive and negative events, and attributing mental conditions to themselves and others (68), which leads to difficulties in recognizing the violation of Grice’s maxims e.g., (40, 69, 70).

In contrast to Language Structure, pragmatic skills assessed by CC-A subscales (Pragmatic Skills and Social Engagement) had no discriminant values. It is possible that the similarities in abilities among the subsamples of participants with disabilities (ability also had no discriminant value), as well as life experience similarities (see the discussion section for the first discriminant function), do not result in greater differences in these scales among ID and DD participants. Also, we should bear in mind that these scales are an indirect method of assessing pragmatic skills, while the ABaCo battery scales are a direct assessment that can be more cognitively demanding for people with DD considering the previously mentioned deficits in executive functions and more pronounced difficulties in linguistic aspects of pragmatics compared to people with ID. In addition, people with DD can have greater difficulties in paralinguistic aspects regarding recognizing expressions on unfamiliar faces (38), which was expected in some tasks of the ABaCo battery, while the CC-A subscales assessed the participants’ pragmatics in usual, everyday communication.

The conversation scale also had no discriminant value. This is a somewhat unexpected result considering that formal thought disorder, which frequently accompanies psychiatric disorders, especially schizophrenia, is manifested in conversational difficulties, such as inappropriately interrupting interlocutors and giving answers irrelevant to the topic (e.g., illogical statements, overly literal or absurd responses, free and indirect associations) (71). The conversation of people with ID and comorbid psychiatric disorders is described as aimless, disorganized, incoherent, and poor (72, 73). Thus, for example, Matson et al. (60) state that communication in people with DD (mild or moderate ID with highly present psychopathological symptoms) is characterized by a poorer ability to elaborate on the initiated topic and recall an earlier conversation. These participants do not know when to stop the conversation respecting the interlocutor’s needs and, thus, often interrupt and/or break the rules when changing the roles of a speaker and interlocutor.

### 5.3. Limitations

The results of this research should be interpreted with caution due to certain limitations. One of the limitations refers to the fact that we did not control the effect of medications in the group of participants with DD. Although it is believed and supported by some research results that language impairments are inherent in schizophrenia and are not the consequence of medications (61), we cannot know to what extent the medications affected the pragmatic skills of these people. Although research studies conducted in Serbia on a sample of adolescents with mild ID indicate low rates of past-month alcohol use compared to the typically developing population, that about 4% have tried marihuana but do not use it regularly, and that the use of cocaine, inhalants, heroin, methamphetamines, ecstasy, steroids, and prescription drugs without a doctor’s prescription was not identified in the present sample (74), future studies should expand the list of exclusion criteria and accordingly control, for example, substance abuse. Since the psychopathological profiles of DD individuals were not measured in this research and the data on symptoms and diagnoses were taken from the participants’ personal records, future research should avoid these mistakes. Also, considering that the socio-economic/educational status of the participants’ parents can be related to the participants’ language abilities, future studies should include the covariates that cover the socio-cultural/socio-economic capital of the family household.

Although the discriminant analysis in our research shows that the ABaCo scale can differentiate all three groups of participants, the initial analyses of Cronbach’s alpha of this instrument indicate that ABaCo is somewhat less reliable in the typically developing group, which should all be taken into account when interpreting the obtained results.

## 6. Conclusion

Pragmatic skills are very complex, and different instruments measure different dimensions of these abilities (e.g., the ABaCo battery and the CC-A questionnaire). The results of this research lead to the conclusion that we can differentiate between the pragmatic abilities of typically developing people, people with ID, and those with DD with the help of the ABaCo battery and the CC-A questionnaire. Participants with DD significantly differ from TD participants in their achievements in two areas – Social Engagement (CC-A) and Paralinguistic scale (ABaCo). On the other hand, participants with ID are significantly more successful than those with DD in the following pragmatic areas – language structure (CC-A), linguistic, paralinguistic, extralinguistic, and context scale (AbaCo).

## Data availability statement

The raw data supporting the conclusions of this article will be made available by the authors, without undue reservation.

## Ethics statement

The studies involving human/animal participants were reviewed and approved by Ethics Committee of the Faculty of Special Education and Rehabilitation from University of Belgrade. The patients/participants provided their written informed consent to participate in this study.

## Author contributions

All authors listed have made a substantial, direct, and intellectual contribution to the work, and approved it for publication.
